# Catatonia in autism spectrum disorders: A systematic review and meta-analysis

**DOI:** 10.1192/j.eurpsy.2021.2259

**Published:** 2022-01-25

**Authors:** J. Vaquerizo-Serrano, G. Salazar De Pablo, J. Singh, P. Santosh

**Affiliations:** 1 Department of Child and Adolescent Psychiatry, Institute of Psychiatry, Psychology and Neuroscience, King’s College London, London SE5 8AF, United Kingdom; 2 Centre for Interventional Paediatric Psychopharmacology and Rare Diseases (CIPPRD), National and Specialist Child and Adolescent Mental Health Services, Maudsley Hospital, London SE5 8AB, United Kingdom; 3 Early Psychosis: Interventions and Clinical-Detection (EPIC) Lab, Department of Psychosis Studies, Institute of Psychiatry, Psychology and Neuroscience, King’s College London, London SE5 8AF, United Kingdom; 4 Institute of Psychiatry and Mental Health, Department of Psychiatry, Hospital General Universitario Gregorio Marañón Instituto de Investigación Sanitaria Gregorio Maranón, Universidad Complutense, Centro de Investigación Biomédica en Red Salud Mental (CIBERSAM), 28009 Madrid, Spain

**Keywords:** ASD, autism, autism spectrum disorders, catatonia, catatonic symptoms

## Abstract

**Background:**

Catatonic features can appear in autism spectrum disorders (ASDs). There can be overlap in symptoms across catatonia and ASD. The overall aim of this review is to provide evidence for the presence of catatonic features in subjects with ASD.

**Methods:**

A systematic literature search using the Web of Science database from inception to July 10, 2021 was conducted following PRISMA, MOOSE guidelines and the PROSPERO protocol. (CRD42021248615). Twelve studies with information about catatonia and ASD were reviewed. Data from a subset was used to conduct meta-analyses of the presence of catatonia in ASD.

**Results:**

The systematic review included 12 studies, seven of which were used for the meta-analysis, comprising 969 individuals. The mean age was 21.25 (7.5) years. Two studies (16.6%) included only children and adolescents. A total of 70–100% were males. Our meta-analysis showed that 10.4% (5.8–18.0 95%CI) of individuals with ASD have catatonia. Motor disturbances were common in ASD subjects with catatonia. No differences were found in comorbidity. Several treatments have been used in ASD with catatonic features, including benzodiazepines, antipsychotics, and electroconvulsive therapy (ECT). The findings of the systematic review showed that ECT might help manage catatonic symptoms.

**Conclusions:**

Different features of catatonia can exist in individuals with ASD and core symptoms of catatonia are reported in ASD. Longitudinal and longer-term studies are required to understand the relationship between catatonia and ASD, and the response of catatonic symptoms to treatment.

## Introduction

Autism spectrum disorders (ASDs) are early onset neurodevelopmental disorders categorized by persistent deficits in social communication and restricted and repetitive patterns of behavior [1]. It is increasingly accepted that ASD are additionally associated with difficulties in sensory processing [2] and motor function [3].

Catatonia, originally described in 1874, is a complex syndrome of abnormal motor, vocal, and behavioral symptoms, with impaired volition and vegetative function [4]. Catatonia has historically been associated with psychosis [5] and was categorized under schizophrenia; however, this condition is now recognized within a range of different disorders, which most commonly occurs in individuals with mood disorders [6]. DSM-5 allows catatonia to be coded as being associated with various mental disorders through the use of a specifier (e.g., neurodevelopmental disorder, brief psychotic disorder, schizophreniform disorder, schizophrenia, schizoaffective disorder, bipolar disorder, major depressive disorder, or other mental disorder) [1]. DSM-5 defines catatonia as being characterized by the presence of at least three of the following symptoms: catalepsy, waxy flexibility, stupor, mutism, negativism, agitation, posturing, stereotypes, mannerisms, grimacing, echolalia, and echopraxia [1] (Supplementary Table S1). Nonetheless, despite the current definition, catatonia remains a poorly recognized condition [6].

There has been increasing interest in the overlap of catatonia and ASD. Symptoms of social indifference, mannerisms, and echolalia are common to both catatonia and ASD [1]. Diagnosis of catatonia in ASD might result in difficulties in its identification due to the overlap in symptoms between these two conditions.

Several explanations for the cooccurrence of catatonia and ASD have been proposed. A prior study has indicated abnormal GABAergic functioning in both conditions when compared to healthy controls [7,8]. Common structural abnormalities in neural circuitry have also been hypothesized [9]. Neuroimaging studies have revealed small cerebellar structures in catatonia and ASD [10]. Furthermore, some authors have suggested a possible genetic connection, with potential susceptibility regions on chromosome 15 implicated in both catatonia and ASD [8,11]. Cases studies of adverse experiences preceding the onset of catatonia also suggest a role for emotional factors [12]. In addition, catatonia has been associated with mood disorders [13], and has been proposed to be an expression of severe anxiety [14]. Susceptibility to anxiety and mood disorders in individuals with ASD might therefore contribute to the high rates of catatonia in this population [15,16].

The presence of catatonic features in ASD has been studied previously [17–22]; nevertheless, to our knowledge, this is the first meta-analysis, that comprehensively assesses the relationship between catatonia and ASD, and quantifies the presence of catatonic features in ASD.

## Methods

This study was done according to the Preferred Reporting Items for Systematic Reviews and Meta-analyses reporting guideline (PRISMA Checklist) [23], as well as the Meta-analysis of Observational Studies in Epidemiology (MOOSE) reporting guideline [24] (Supplementary Table S2). The study protocol was registered in PROSPERO (CRD42021248615).

### Search strategy and selection criteria

A multistep search of the literature was undertaken by two independent researchers (J.V.S and G.S.P.) through the Web of Science database (Clarivate Analytics), incorporating the Web of Science Core Collection, BIOSIS Citation Index, KCI-Korean Journal Database, MEDLINE, Russian Science Citation Index, SciELO Citation Index, Cochrane Central Register of Reviews, and Ovid/PsychINFO databases from inception until July 10, 2021, using the following keywords: “Catatonia” OR “Catatonic” OR “Catatoni*” AND “Autism” OR “Autism Spectrum Disorders” OR “Autistic Disorder” OR “ASD.”

Preprint servers “medRxiv” and “PsyArXiv” were also searched from inception until July 10, 2021, using the keywords *“Catatonia” AND “Autism.”* To supplement the search, the references of systematic reviews or meta-analyses that were retrieved were also manually searched. Following the screening out of the abstracts of articles identified deemed not relevant, the remaining full-text articles were then assessed against the eligibility (inclusion and exclusion) criteria.

### Eligibility criteria

#### Inclusion criteria

The included studies were: (a) individual studies, including abstracts, conference proceedings, or gray literature (i.e., “medRxiv” and “PsyArXiv”); (b) in (i) individuals with ASD in whom the presence of catatonia is reported; (ii) individuals with catatonia in whom the presence of ASD is reported, and (iii) in which the overlapping and/or distinctive features between catatonia and ASD are described, providing important data on the relationship between them; and (c) published in English.

Additional inclusion criteria were used for the meta-analysis: (a) reporting meta-analyzable data, and (b) nonoverlapping samples. As described above overlap between the included studies was actively searched by evaluating the country, setting, university, and program from which the study sample was obtained. The recruitment period was also examined. If there was a case, when more than one study from the same sample was identified, the study with the largest sample was included.

#### Exclusion criteria

The following exclusion criteria used were: (a) reviews, clinical cases, and study protocols; (b) studies that did not formally assess and select participants with catatonia or ASD, and (c) studies written in languages other than English.

### Outcome measures and data extraction

Independent data extraction was performed by two researchers (J.V.S. and G.S.P.), and discrepancies were resolved through discussions with the senior author (P.S.). The following variables were extracted: Study (first author and year of publication); Program; City; Country; Setting; Recruitment Period; Study Type (original or abstract); Design of the Study (longitudinal or cross-sectional); Main Outcome; Topic Investigated; Diagnoses; % ASD; % Catatonia; Comparison Group (if available); Sample Size; Age (mean, SD); Sex (% Males); Age of Onset; Diagnostic Instrument; Comorbidity (if applicable); Treatment Received (if applicable); Key Findings, and Quality Assessment.

### Quality assessment

Study quality was evaluated in all the included studies. Although quality assessments can be conducted in meta-analyses, their use in observational studies is controversial, with no clear agreement on rating methods or their use in the analysis [25].

The quality assessment was performed using a modified version of the Newcastle-Ottawa Scale for the evaluation of longitudinal and cross-sectional studies, (www.ohri.ca/programs/clinical_epidemiology/oxford.asp) [26], in line with prior meta-analyses [27]. Scores ranged from 0 to 8 (Supplementary Table S3).

### Data synthesis and meta-analysis

In this study, the existing evidence on the relationship between catatonia and ASD was systematically reviewed. We focused on overlapping features, including clinical, therapeutic, and cognitive aspects. When reporting the data, the results were extracted according to the type of study, that is, the data described at the baseline were obtained from cross-sectional studies and the characteristics developed over time were obtained from longitudinal studies. The presence of catatonia in individuals with ASD (%, Standard Error) was the primary outcome.

Due to the notion that the studies in this meta-analysis were expected to be heterogeneous, the random-effects model was used [28]. Heterogeneity between studies was measured with the *Q* statistic and its magnitude was evaluated with the *I*-squared index [29]. We performed a sensitivity analysis, stratified by group that evaluated the design of the study to determine whether there were differences between cross-sectional and longitudinal studies. Publication bias was assessed by visually inspecting funnel plots [30] and applying the regression intercept of Egger [31]. Due to the scarcity in the number of evaluable studies, a test of moderating factors using meta-regression analysis to estimate sources of heterogeneity could not be performed. All *p*-values reported in the meta-analysis were two-sided and the level of significance was set at a *p*-value of less than 0.05. Comprehensive Meta-analysis Software, version 3 (Biostat, Inc., Englewood, NJ) [32] was used.

## Results

### Database

**Figure 1.**
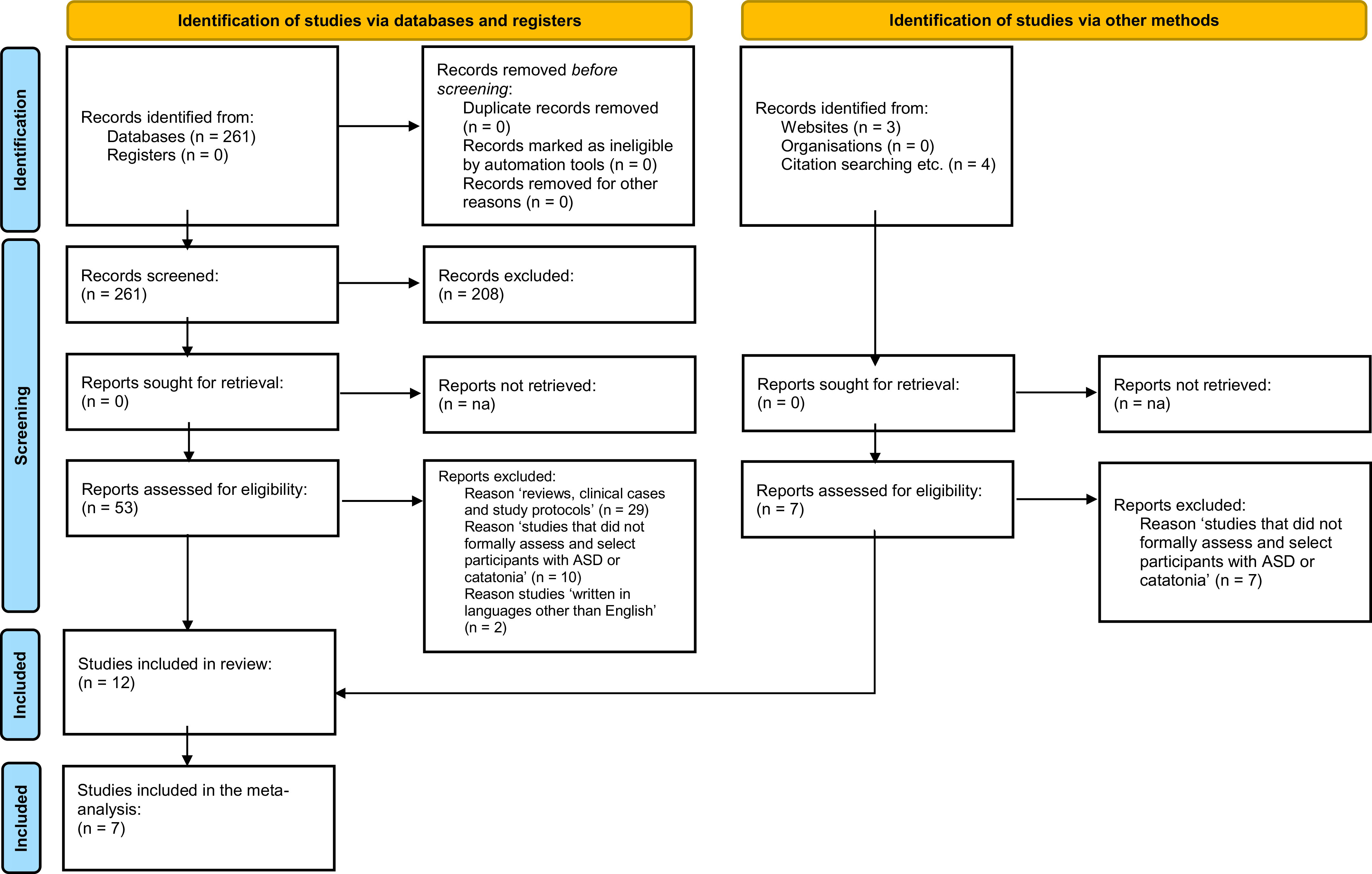
PRISMA flowchart.

The literature search returned 268 citations that were screened for eligibility. Of those, 215 were excluded during the title and abstract screening, and overall 53 full-text articles were assessed for eligibility. This step resulted in a total of 12 studies being included in the current systematic review, which included a total of 1,534 individuals. After excluding overlapping samples, and those studies that did not provide meta-analyzable data, seven studies comprising 969 individuals, were included in the meta-analysis on the prevalence of catatonia in ASD (Figure 1).

### Study characteristics

The characteristics of the included studies are detailed in Table 1. Four studies (33.3%) were from the US [33–36], seven (58.3%) from Europe [37–43] and one (8.3%) from Asia [44]. All 12 studies reported data on general characteristics of catatonia and ASD.

**Table 1.** Main characteristics of the included studies regarding catatonia and ASD.

Study	Study type and design (NOS)	Type of sample	Country	Sample	Diagnostic criteria	Mean age (SD)	Gender %Males	Key findings
Billstedt et al. [37]	Longitudinal (6)	Population-based	Sweden	108 ASD	DSM-III/DSM-IV/ICD-10/HBSS/CARS/ABC/Wing criteria/DISCO	25.5	70	ASD subjects showed a poor outcome at follow-up. Childhood IQ-level was positively correlated with better adult outcome, as was the existence of some communicative phrase speech at age 6 years. Those diagnosed with catatonia had severe motor initiation problems
				13 Catatonia				
Breen and Hare [38]	Cross-sectional (5)	Population-based	UK	99 ASD	ABQ	15.7	79.8	Attenuated behavior indicative of catatonia was common in young people with ASD. Six catatonic core symptoms were commonly reported, with difficulty initiating movement being least reported and physical and/or verbal prompts required most reported
				20 Catatonia				
Ghaziuddin et al. [33]	Cross-sectional (7)	Clinic-based	US	81 ASD	Clinical criteria	13.9 (2.04)	80.2	Subjects with catatonia were older and had a lower score of global functioning. Aggression was more common among the group diagnosed with catatonia compared with the noncatatonic group. Similarly, reduced movements and loss of speech were also more common among this group
				18 Catatonia				
Hare et al. [45]	Cross-sectional (5)	Population-based	UK	99 ASD	ABQ	15.7	79.8	Catatonia appears to be more prevalent in both ASD and in genetic syndromes that are strongly associated with comorbid ASD than was previously thought
				20 Catatonia				
Hutton et al. [40]	Longitudinal (5)	Population-based	UK	135 ASD	ADI-R/ADOS/SAPPA/ICD-10	na	77	The presence of obsessive–compulsive behavior and of catatonia, which mainly seemed to stem from obsessive–compulsive symptoms, might be particularly characteristic of ASD. Of 135 individuals with ASD, 16% reported a new psychiatric disorder, of whom three patients reported a catatonia
				3 Catatonia				
Ohta et al. [44]	Longitudinal (4)	Clinic-based	Japan	11 ASD	DSM-IV/ICD-10/Wing criteria	27.6 (5.5)	100	The average age at onset of catatonia was 19 years (ranged 15–23). A total of 11.6% ASD had catatonia. Before the manifestation of a typical catatonic symptom, eight cases had prodromal symptoms, typically a gradually emerging lethargy with compulsive behaviors lasting for more than 1 year
				2 Catatonia				
Périsse et al. [43]	Cross-sectional (6)	Clinic-based	France	29 ASD	ICD-10 /CARS	14.8 (1.3)	79.3	Disruptive behaviors among adolescents with ASD may stem from diverse risk factors, including environmental problems, comorbid acute psychiatric conditions, or somatic diseases
				2 Catatonia				
Wachtel [34]	Longitudinal (4)	Clinic-based	US	22 ASD	BFCRS	8–25	72.72	Multiple symptoms of catatonia are found in patients with ASD. Some of the most severe presentations include psychomotor excitement in the form of repetitive self-injury. The use of short-term and maintenance ECT might be helpful
				18 Catatonia				
Wachtel [36]	Longitudinal (4)	Clinic-based	US	22 ASD	DSM-5	8–26	72.72	The most common catatonic symptoms were agitation, stereotypy, posturing, negativism, mutism, stupor and grimacing. 91% ASD patients displayed repetitive self-injury
				22 Catatonia				
Wachtel [35]	Longitudinal (5)	Clinic-based	US	22 ASD	DSM-5	8–26	72.72	Benzodiazepines were of unclear benefit in the resolution of catatonia in ASD. Reasons for discontinuation included no benefit in terms of catatonic symptom reduction (31.8%), partial catatonic symptom reduction (40.9%), sedation (13.6%), and behavioral worsening (9.1%). A total of 76% patients who completed a trial of lorazepam, demonstrated catatonic symptom resolution. Four patients who failed lorazepam therapy responded to ECT. Maintenance electroconvulsive therapy was necessary for sustained symptom remission
				22 Catatonia				
Wing and Shah [41]	Cross-sectional (7)	Clinic-based	UK	506 ASD	ICD-10/DISCO/Wing criteria	24.9	82	Catatonia onset ranged 10–19 years. Precipitating factors were suggested. From the onset of catatonia, 10% experienced a slow but steady deterioration in mobility and practical skills. Most of the abnormalities of movement resulted in slowing or stopping activities. There was an excess of males, more marked among those with catatonia. A total of 50% with catatonia-like deterioration were passive in social interaction. Catatonia was seen in a higher proportion of those with learning disabilities and language impairment
				30 Catatonia				
Wing and Shah [42]	Cross-sectional (7)	Clinic-based	UK	200 ASD	DISCO/Wing criteria	12.7 (8.1)	83.5	The most frequent catatonic symptoms reported from the DISCO were lack of facial expression, delayed echolalia, odd intonation, lack of cooperation, and poor eye contact. The lowest frequencies were found among those concerning fascination with visual stimuli. Higher IQ tended to be associated with less marked manifestations of the item concerned
				7 Catatonia				
				36 ASD		2.8–11.6	72	ASD was rated with more marked problems than the other three groups on DISCO. More children with learning disability had marked or moderate problems than those with specific language impairment, except for delayed echolalia, noisiness, and aggressiveness, which happened more often in those with specific language impairments. ASD individuals displayed more anxiety symptoms, more relevant in those with IQ > 70
				17 Learning disabled				
				14 Language impaired				
				15 HC				
				506 ASD		24.9	82	A total of 6% individuals with ASD had catatonia, 17% of those were > 15 years. Impairment of social interaction was one of the main outcomes. 50% with catatonia were passive in social interaction and were also more impaired in expressive language
				30 Catatonia				

Abbreviations: ABC, the Autistic Behavior Checklist; ABQ, the Attenuated Behavior Questionnaire; ADI-R, the Autism Diagnostic Interview-Revised; ADOS, Autism Diagnostic Observation Schedule; ASD, Autism Spectrum Disorder; BFCRS, Bush–Francis Catatonia Rating Scale; CARS, the Childhood Autism Rating Scale; DISCO, the DIagnosis of Social and COmmunication Disorder Schedule; DSM-III, Diagnostic and Statistical Manual of Mental Disorders, third edition; DSM-IV, Diagnostic and Statistical Manual of Mental Disorders, fourth edition; DSM-5, Diagnostic and Statistical Manual of Mental Disorders, fifth edition; ECT, electroconvulsive therapy; HBSS, the MRC Handicaps, Behavior & Skills (HBS) schedule; HC, healthy control; ICD-10, International Classification of Diseases 10th Revision; NOS, Newcastle-Ottawa Scale for the evaluation of longitudinal and cross-sectional studies; SAPPA, the Schedule for Assessment of Psychiatric Problems Associated with Autism.

The mean age across the 12 included studies was 21.25 (7.5) years, ranging from 12.7 to 27.6 years. Two studies (16.6%) included only children and adolescents [33,43]. Most of the studies had a higher percentage of males, (range between 70 and 100% of the total sample) (key findings in Supplementary Table S4).

### Clinical characteristics of individuals with ASD and catatonic features

The systematic review showed that 20.2% of ASD individuals had features of catatonia [38,45]. Of those ASD individuals with catatonic features, 85% had motor disturbances [38]; 5.7–81.6% had an intellectual disability (ID) [37,38,40], and 14.1–46.6% of those had severe impairment [37,40]. A total of 34.2% had language problems, with poorer functioning being associated with a lack of phrase speech during early childhood [37].

In terms of catatonic symptomatology: (a) impaired speech was present in 29.0–100% [33,41]; (b) lack of cooperation and negativism was present in 69.5–85.0% [42]; (c) agitation uninfluenced by external stimuli in 62.0–75.2% [33,42]; (d) aggression was reported in 62.0–70.3% [33,42]; (e) posturing was informed in 63.3% of individuals [41]; (f) echolalia was present in 47.5–61.3% [42]; (g) grimacing in 54.0–55.6% [42]; (h) stereotypies and other repetitive movements were present in 19.4–61.1% [41,42]; (i) 30% had odd social communication and difficulty in identifying emotions or experiences [41], and (j) 50% of the ASD individuals with catatonic features were passive in social interactions [41,42].

### Comorbid psychopathology in individuals with ASD and catatonic features

Comorbid psychopathology in ASD individuals with catatonic features included anxiety in 22.2–69.45% [42], of whom 39–83% had marked anxiety [42]; obsessive–compulsive traits were present in 26.6% [41], and 44.0–55.6% had hyperactivity [42]. Further, 11.1–13.0% had epilepsy [33,41]. Importantly, ASD subjects with more core catatonic features had significantly more depressive symptoms [38,45].

### Clinical characteristics of ASD individuals who developed catatonic features during follow-up

During the follow-up of ASD individuals, 2.2–12.0% developed catatonic features [37,40]. The catatonic symptoms described were: (a) agitation in 18.2–95.5% [33,36,41,42]; (b) stereotypies in 90.1% [36]; (c) posturing in 81.8% [36]; (d) negativism in 77.3% [36]; (e) mutism in 63.6% [36]; (f) grimacing in 31.8% [36]; (g) echolalia in 9.1% individuals [36]; (h) aggression in 18.2–19.0% [44], and (i) self-harming behaviors in 27.7–90.9% [36,37,44]. In addition, 71% had severe intellectual disability [37] and 3.7–12.0% present severe motor initiation problems at follow-up [37].

### Clinical comorbidity of ASD individuals who developed catatonic features during follow-up

In ASD individuals who developed catatonic features, 60.0–72.7% [40,44] reported obsessive–compulsive symptoms. A total of 9.1% had depression, adjustment disorder and sleep disturbances [40]. A total of 9.1–33.0% reported hyperactivity [37,44], and 27% had Tourette syndrome [44]. In addition, 27% had epilepsy [44].

### Interventions for catatonic features in ASD

Several treatments have been used in ASD with catatonic features. Our review found that antipsychotics were used in 27–100% of individuals [33] and benzodiazepines were used in 55.6–95.5% [35]. Electroconvulsive therapy (ECT) was used in 22 individuals out of 1,534 [34,35]. The systematic review showed that ECT improved catatonic symptoms, but benzodiazepines did not show a clear benefit in the resolution of catatonia, with benzodiazepines being discontinued in 33.3% due to lack of improvement; 38.1% due to only partial response; 9.5% due to sedation; and 9.5% because of behavioral worsening [35].

### Results of the meta-analysis

**Figure 2.**
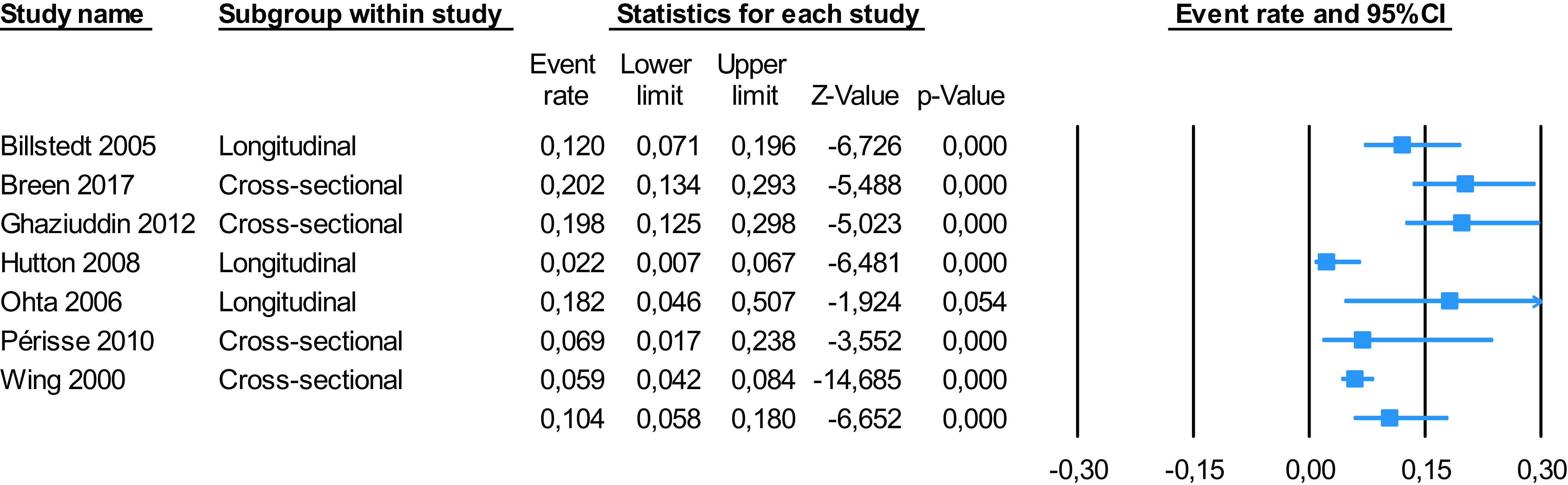
Meta-analysis. Catatonia in autism spectrum disorder.

Seven studies had data that allowed meta-analysis, comprising 969 individuals [33,37,38,40,41,43,44]. Overall, the meta-analytical results show that 10.4%, (5.8–18.0 95%CI) of individuals with ASD have catatonia (Figure 2). Heterogeneity was significant (*Q* = 36.597, *I* ^2^ = 83.605%). Egger’s test result did not reveal significant publication bias (*t* = 0.018, *p* = 0.986) (Supplementary Figure S1 and Supplementary Table S5).

Sensitivity analyses stratified by group, (cross-sectional vs. longitudinal studies), revealed that 12.1%, (5.5–24.6 95%CI) of individuals with ASD have catatonia cross-sectionally, and 8.0% (2.4–23.4 95%CI) of subjects diagnosed with ASD develop catatonic symptoms during the follow-up. There were no statistical differences in prevalence between cross-sectional and longitudinal studies (*p* = 0.801).

### Quality assessment

The quality assessment of the included studies, evaluated using a modified version of the Newcastle-Ottawa Scale, was 5.41 ± 1.24 and ranged from 4 [34,35,44] to 7 [33,36–38,40–43,45].

The full results are detailed in Supplementary Tables S6 and S7.

## Discussion

This is the first meta-analysis that comprehensively addresses the presence of catatonia in ASD. We systematically reviewed 12 studies which had information about both ASD and catatonic features, allowing us to explore the relationship between these two conditions. In addition, we meta-analytically addressed the presence of catatonia in ASD.

Individuals with catatonia and ASD present as a heterogeneous group. The present study demonstrates that these individuals are young, (mean age range 12.7–27.6 years), with catatonia having an onset in late adolescence in ASD, with a peak between 15 and 19 years [41,42,44]. Furthermore, according to our findings, catatonia appears more frequently in males, (70–100%), which is also concordant with previous studies [46,47].

Our meta-analysis revealed that 10.4%, (5.8–18.0 95%CI) of individuals with ASD have catatonia, which is in keeping with the results from the review, and not too dissimilar from the 9.0% prevalence of catatonia in a variety of psychiatric or medical conditions [48]. However, this was reported to be lower (7.8%) in a subgroup with low heterogeneity [48]. Sensitivity analyses stratified by group revealed no differences depending on the type of study, meaning that we could consider the overall sample included in both cross-sectional and longitudinal studies to calculate the presence of catatonia in ASD.

The symptom overlap between catatonia and ASD have been clearly described (Supplementary Table S8). The differentiating feature is that the symptoms in catatonia are typically new-onset (usually during late adolescence) or due to a considerable worsening of existing symptoms, unlike ASD, which starts in early preschool years. Catatonia and ASD are well-known to exhibit symptom overlap, including mutism, negativism, abnormal speech, echolalia, posturing, grimacing, stereotypies, mannerisms, and purposeless agitation [21,49]. Motor abnormalities, mannerisms, and stereotypies are present in ASD independent of the cooccurrence of catatonia [50,51], and a recent meta-analysis revealed that the prevalence of stereotypies in ASD was around 51.8%, with a range between 21.9 and 97.5% [51].

When intelligence is considered, 70–75% of ASD were traditionally estimated to have intellectual disability (ID) [52]; however, recent estimates suggest that only 50–55% of ASD individuals have ID [53]. In our systematic review, the rate of ID in ASD with catatonia ranged between 5.7 and 81.6% [37,38,40], reaching 100% when considering clinic-based studies [35,41,42,44]. Nonetheless, those studies have clinical populations and use older definitions of ASD, with very high comorbidity of ID, which does not represent the current frequency of ID in ASD.

When clinical symptomatology at presentation is reviewed from the perspective of both clinic and population-based studies, our systematic review showed that the proportion of ASD with catatonia have a history of speech reduction is somewhat higher (29.0–100% vs. 42.9%) compared to that reported in previous studies [54]. Literature highlights the presence of mutism (up to 97.0%) [54–57] and negativism (ranging between 59.5 and 85.0%) [42,54] in individuals with catatonia. Agitation uninfluenced by external stimuli was reported between 62.0 and 75.2%, which is similar to previous studies, where agitation was reported in 64.3% of catatonic individuals [54]. Considering the literature, where stupor and negativism are more frequently reported in earlier studies [58,59], there might be a lack of recognition of the agitated subtype of catatonia. Stereotypies and posturing were reported in 90.1 and 81.8%, respectively [36], but other studies have reported lower figures of 35.7% for stereotypies and 19.0% for posturing [54]. Stupor and immobility have been reported as common symptoms in catatonia [6,57]. In the longitudinal data that we examined, agitation was the commonest catatonic symptom in those with ASD who developed catatonia, reported in 18.2–95.5%. Further, self-harming behaviors were observed up to 90.9%. It is well documented that self-injury and aggression are often seen in catatonia [37,49,60] and ASD [61].

The presence of obsessive–compulsive symptoms and catatonia appears to be particularly characteristic in ASD individuals [40]; however, the relationship between both is still generally misunderstood [62]. Obsessive–compulsive symptoms were found in 26.6–72.7% preceding catatonic symptoms [41,44]. At the symptomatic level, it has been described that patients with obsessive–compulsive disorder might present catatonic features as a direct result of their obsessive–compulsive symptoms. Especially obsessive slowness and counting rituals can present as catatonic symptoms. Catatonia has also been reported to occur more frequently in people with mood disorders [13]. Prior studies have found that mood disorders appeared between 36.0 and 63.2% in catatonic individuals [54,63,64]. Our review showed that 9.1% of ASD individuals with catatonia had depression, a much lower rate compared to previously reported. This discrepancy might be due to the heterogeneity of the diagnostic criteria, and the lower rates of recognition of depression in ASD. Further, tics and Tourette’s syndrome have been described to be comorbid with ASD in several studies [65,66], and are also seen in catatonia [49]. In our review, 27% ASD subjects with catatonia also had Tourette syndrome; however, in a prior study, 87% of patients with Tourette’s syndrome described the presence of catatonic symptoms [67]. Surprisingly, tics have not regularly been described in catatonia, which may appear unusual as repetitive movement abnormalities are considered classic symptoms of catatonia [68].

Varied treatments have been tried to treat catatonia. Our systematic review revealed that in catatonia with ASD, antipsychotics were very frequently used (27–100%) [33,34], and that the catatonia did not clearly respond to benzodiazepines, and often had to be stopped because of treatment-induced side effects [35]. This pharmacological profile significantly differs from the treatment response reported in catatonia generally, where it is accepted that there is no evidence for the use of antipsychotics in catatonic patients without an underlying psychotic disorder [69]. There is support for the efficacy of benzodiazepines, especially lorazepam, in mild catatonia in typically developing individuals associated with affective symptoms when treatment is initiated quickly after symptom onset [59,70]. Further, benzodiazepines are the most extensively studied treatment, with reports of good response and good tolerability [69]; Our review suggests that catatonic patients with ASD may respond less robustly to benzodiazepines, based on a retrospective chart review of inpatient and outpatient clinical records in 22 ASD patients being treated for catatonia [35]. Although the underpinnings of treatment response are unclear in catatonia with and without ASD, catatonia in ASD might have distinctive underlying deficits that might make it less responsive to certain treatments such as benzodiazepines. Some literature suggests that GABA dysfunction appears to be a common biological substrate in both [13,49]. With regard to antipsychotic treatment, its use should be carefully considered [71]. Some authors recommend avoiding antipsychotics altogether in catatonic patients, due to the risk of worsening the condition or even inducing malignant catatonia [57,72,73]; however, this unfavorable effect is especially associated with the use of first-generation antipsychotics [74]. Nevertheless, low doses of atypical antipsychotics are known to have weak γ-aminobutyric acid agonist activity and serotonin antagonism, that could stimulate dopamine release in the prefrontal cortex and thus alleviate catatonic symptoms [75]. There are case reports of successful treatment with atypical antipsychotics [71,76,77]. While this information is useful, further work is needed in larger samples to further gauge the usefulness of different treatments.

### Limitations

The small number of studies appraised and the differences in diagnostic criteria used within these studies limits the generalizability of our findings. This is further confounded because at present there is no gold-standard measure used for the identification of catatonia features in autism. Furthermore, the quality appraisal showed that the quality of most of the included studies was low. In addition, when addressing catatonia, most studies in the previous literature were case reports and clinic-based studies, which makes it difficult to extrapolate and infer clinically meaningful findings.

Despite the total number of participants included in the current meta-analysis is large (*n* = 969 from 7 studies), and the results are significant with precise 95% CIs to evaluate the presence of catatonia in ASD, due to the lack of data in publications, our meta-analysis did not allow us to do meta-regression analyses to examine the relationship of characteristics such as clinical, psychopathological, therapeutic, cognitive, and neurobiological aspects within these two conditions. Likewise, we were unable to analyze the presence of other comorbid conditions in individuals with autism and catatonia.

Moving forward, longer-term studies would be required to evaluate the overlap between catatonia and ASD regarding social, volitional, verbal, and motor impairments. Additionally, longitudinal studies are required to investigate the relationship between catatonia and ASD, especially treatment response. Furthermore, studies that measure anxiety, low mood, motor impairment, anhedonia, mutism, stressful life events, and treatment response in catatonia with and without ASD will help to further our understanding on the different features in both of these conditions. Timely assessment and intervention in individuals with catatonia and ASD offer more scope to improve upon current treatment pathways.

## Conclusions

Different features of catatonia can exist in individuals with ASD. Core symptoms of catatonia are reported in ASD. Motor abnormalities, mannerisms and stereotypies are present in both conditions. From a clinical perspective, the neurobiological overlap between catatonia and ASD might make early intervention and treatment more difficult. Longitudinal studies are required to investigate the relationship between catatonia and ASD, and to explore treatment response to antipsychotics and benzodiazepines in catatonia with and without ASD.

## Data Availability

The data that support the findings of this study are available from the corresponding author upon reasonable request.

## Supplementary Materials

To view supplementary material for this article, please visit http://doi.org/10.1192/j.eurpsy.2021.2259. click here to view supplementary material
