# Supplementary material for: Catatonia in autism spectrum disorders: A systematic review and meta-analysis
Source: Eur Psychiatry. 2022 Jan 25;65(1):e4. doi: 10.1192/j.eurpsy.2021.2259 (PMC8792870; doi:10.1192/j.eurpsy.2021.2259)
Supplement: Supplementary file 1 [file S0924933821022598sup001.docx]

**CATATONIA IN AUTISM SPECTRUM DISORDERS:**

**A SYSTEMATIC REVIEW AND META-ANALYSIS.**

**SUPPLEMENTARY MATERIAL**

- **eTable 1:** Diagnostic criteria for catatonia.
- **eTable 2:** MOOSE checklist.
- **eTable 3:** Risk of bias (quality) assessment using the modified Newcastle Ottawa Scale for cross-sectional and longitudinal studies.
- **eTable 4:** Key Findings.
- **eFigure 1:** Meta-funnel results for Catatonia in ASD
- **eTable 5:** Meta-analytical results. % Catatonia in ASD.
- **eTable 6:** Risk of bias (quality assessment) using modified Newcastle Ottawa Scale for longitudinal studies.
- **eTable 7:** Risk of bias (quality assessment) using modified Newcastle Ottawa Scale for cross-sectional studies.
- **eTable 8:** Symptom overlap between Catatonia and ASD.

This supplementary material has been provided by the authors to give readers additional information about their work.

**eTable 1.** Diagnostic criteria for catatonia.

|  | DSM-IV (1) | DSM 5 (2) | ICD-10 (3) |
| --- | --- | --- | --- |
| Diagnostic Criteria | At least one for Catatonia secondary to a general medical condition, or two for Catatonic schizophrenia or specifier of mood disorder of the following:   1. Motor immobility as evidenced by catalepsy, including waxy flexibility, or stupor 2. Excessive motor activity, that is apparently purposeless and not influenced by external stimuli 3. Extreme negativism, resistance seemingly no reason at all commands or maintenance of a rigid posture against attempts to be moved, or mutism 4. Peculiarities of voluntary movement as evidenced by the trend towards fixed posture, stereotyped movements, mannerisms or prominents grimacing 5. Echolalia or echopraxia | Three or more of the following:   1. Catalepsy (passive induction of a posture held against gravity) 2. Waxy flexibility (slight and even resistance to positioning by examiner) 3. Stupor 4. Agitation, not induced by external stimuli 5. Mutism 6. Negativism (opposing or not responding to instructions or external stimuli) 7. Posturing (spontaneous and active 8. maintenance of a posture against gravity) 9. Mannerisms (odd caricature of normal actions) 10. Stereotypies (repetitive, abnormally frequent, non-goal- directed movements) 11. Grimacing 12. Echolalia (mimicking another’s speech) 13. Echopraxia (mimicking another’s movements) | Catatonic schizophrenia: General criteria for schizophrenia are met. For at least two 2 weeks, the presence of 1 or more of the following:   1. Stupor or mutism 2. Excitement (apparently purposeless motor activity, not influenced by external stimuli) 3. Posturing (voluntary assumption and maintenance of inappropriate or bizarre postures) 4. Negativism (an apparently motiveless resistance to all instructions or attempts to be moved, or movement in the opposite direction) 5. Rigidity (maintenance of a rigid posture against efforts to be moved) 6. Waxy flexibility (maintenance of limbs and body in externally imposed positions) 7. Command automatism (automatic compliance with instructions).   Organic catatonic disorder: A disorder of diminished (stupor) or increased (excitement) psychomotor activity associated with catatonic symptoms in the context of an organic brain disorder. The extremes of psychomotor disturbance may alternate. |
|  | **DSM-IV** | **DSM 5** | **ICD-10** |
| Catatonia diagnosis | Catatonic schizophrenia  Specifier major mood disorders | Catatonic disorder due to a general medical condition  Catatonia specifier for   1. Schizophrenia 2. Schizoaffective disorder 3. Schizophreniform disorder 4. Brief psychotic disorder 5. Substance-induced psychotic disorder   Catatonia specifier for affective disorders   1. Major depressive disorder 2. Bipolar I disorder 3. Bipolar II disorder 4. Catatonic disorder NOS | Catatonic schizophrenia  Organic catatonic disorder |

**eTable 2:** MOOSE checklist. (4)

| Criteria | | Brief description of how the criteria were handled in the meta-analysis |
| --- | --- | --- |
| Reporting of background should include | |  |
| √ | Problem definition | No meta-analysis has evaluated the presence of catatonia in ASD |
|  | Hypothesis statement |  |
| √ | Description of study outcomes | Studies were described in the supplementary table |
|  | Type of exposure or intervention used |  |
| √ | Type of study designs used | Both cross sectional and longitudinal studies were selected |
| √ | Study population | Subjects with catatonia and ASD |
| Reporting of search strategy should include | |  |
| √ | Qualifications of searchers | The credentials of the investigators are indicated in the author list and in the acknowledgements |
| √ | Search strategy, including time period included in the synthesis and keywords | We performed a multi-step literature search using keywords described in methods section: until 21st April 2021 |
| √ | Databases and registries searched | Web of Science database (Web of Science Core Collection, BIOSIS Citation Index, KCI-Korean Journal Database, MEDLINE, Russian Science Citation Index, and SciELO Citation Index) and grey literature |
| √ | Use of hand searching | References of systematic reviews or meta-analyses that were screened during literature search and the references from the included studies were manually searched |
| √ | List of citations located and those excluded, including justifications | Details of the literature search process are outlined in the results section and PRISMA flowchart |
| √ | Method of addressing articles published in languages other than English | Only articles in English language were selected |
| √ | Method of handling abstracts and unpublished studies | Original individual studies were included. Reviews, clinical cases and study protocols were excluded |
| √ | Description of any contact with authors | We did not contact authors |
| Reporting of methods should include | |  |
| √ | Description of relevance or appropriateness of studies assembled for assessing the hypothesis  to be tested | Detailed inclusion and exclusion criteria are described in the methods section |
| √ | Rationale for the selection and coding of data | Data extracted from each of the studies are relevant to the population characteristics, study design and studies outcomes |
| √ | Assessment of confounding | We did not investigate confounding factors as stated in the limitations section |
| √ | Assessment of study quality, including blinding of quality assessors; stratification or regression on possible predictors of study results | We evaluated the quality using Mixed Methods Appraisal tool |
| √ | Assessment of heterogeneity | Heterogeneity was assessed with the I^2^ index |
| √ | Description of statistical methods in sufficient detail to be replicated | A random-effects meta-analysis was used. Heterogeneity among study point estimates was assessed using Q statistics. The proportion of the total variability in the effect size estimates was evaluated with the I^2^ index |
| √ | Provision of appropriate tables and graphics | We included the PRISMA flow-chart to describe the literature search and its results |
| Reporting of results should include | |  |
| √ | Table summarizing individual study estimates and overall estimate | We reported this in the results |
| √ | Table giving descriptive information for each study included | We have presented descriptive information for each study in the tables and as supplementary material |
| √ | Results of sensitivity testing | We did not conduct subgroup analyses |
| √ | Indication of statistical uncertainty of findings | We reported this in the results section |
| Reporting of discussion should include | |  |
| √ | Quantitative assessment of bias | Publication biases was assessed using a modified version of the Newcastle-Ottawa Scale was used for cross-sectional and longitudinal studies. Scores ranged from 0 to 8 |
| √ | Justification for exclusion | We excluded studies based on the rationale of the meta-analysis |
| √ | Assessment of quality of included studies | The quality of the studies was assessed and reported |
| Reporting of conclusions should include | |  |
| √ | Consideration of alternative explanations for observed results | We have addressed this point in the discussion section |
| √ | Generalization of the conclusions | We have addressed this point in the discussion section |
| √ | Guidelines for future research | We have addressed this point in the discussion section |
| √ | Disclosure of funding source | Funding source was specified |

**eTable 3**: Risk of bias (quality) assessment using the modified Newcastle Ottawa Scale (NOS) for cross-sectional and longitudinal studies.

| Criteria | Maximum Score |
| --- | --- |
| *Cross-Sectional Studies* | |
| Sample representative of target sample (e.g., all eligible or random sample)? | 2 |
| Sample size justified and satisfactory? | 1 |
| Non-response rate is defined, satisfactory, and characteristics of responders/non-responders compared? | 1 |
| Ascertainment of exposure is valid and/or well-described? | 1 |
| Assessment of outcome with robust tool and/or record linkage? | 2 |
| Outcome per group reported appropriately? | 1 |
| *Cohort Studies* | |
| Representativeness of exposed cohort (e.g. total population or random sample, selected group) | 1 |
| Method used to ascertain exposure is robust? | 1 |
| Exposed and unexposed are matched or adjustment for confounding factors? | 2 |
| Assessment of outcome was blind to exposure status or used record linkage, were robust tools used? | 2 |
| Follow-up period was sufficiently long for outcomes to occur? | 1 |
| Loss to follow-up rate is reported, low (<30%), and same in exposed and non-exposed? | 1 |

**eTable 4.** Key Findings.

| Autism Spectrum Disorder (ASD) & Catatonia | Systematic Review Findings at Baseline | Systematic Review Findings at Follow-up | Metaanalysis Results |
| --- | --- | --- | --- |
| - Studies: 12 - N=1,534 individuals. - Mean age: 21.25 (7.5) years, ranging from 12.7 to 27.6 years. - Males: 70-100% | - Presence of catatonia in ASD was 20.2%. - 5.7%–81.6% had an intellectual disability, 14.1%-46.6% of whom had severe impairment. 34.2% of ASD individuals had language problems. 85% had motor disturbances. Impaired speech was present in 29.0%-100%. - Negativism was present in 69.5%-85.o%. Agitation was reported between 62.0%-75.2%. Aggression between 62.0% and 70.3%. Posturing in 63.3%. Echolalia ranged between 47.5%-61.3%. Grimacing was present in 54.0%-55.6%. Stereotypies in 19.4%-61.1%. - 30% had odd social communication and difficulty in identifying emotions or experiences. 50% were passive in social interactions. - Obsessive-compulsive symptoms were found in 26.6% preceding catatonia. Anxiety was reported in 22.2%-69.45%, of whom 39%-83% had marked anxiety. 44.0%-55.6% showed hyperactivity symptoms. Between 11.1%-13.0% had epilepsy. | - Catatonia in ASD was reported between 2.2-12.0%. - The rate of severe intellectual disability was reported in 71%. Between 3.7%-12.0% had severe motor initiation problems. - Agitation was present in 18.2%-95.5%. Stereotypies in 90.1%. Posturing in 81.8%. Negativism in 77.3%. Mutism in 63.6%. Grimacing in 31.8%. Echolalia was described in 9.1%. Aggression in 18.2-19.0%. Self-harming behaviours were observed in 27.7%-90.9. - Between 60.0% and 72.7% of ASD individuals with obsessive-compulsive symptoms developed catatonic features. 9.1% - 33.0% of ASD individuals with catatonic features showed hyperactive behaviours. Both Tourette syndrome and epilepsy were present in 27.0%. Depression, adjustment disorder and sleep disturbances were reported in 9.1%. | - Studies: 7 - N= 969 individuals. - 10.4%, (5.8-18.0 95%CI) of individuals with ASD have catatonia. - Heterogeneity was significant (Q=36.597, I2=83.605%). - Egger's test result did not reveal significant publication bias (p=0.986) - Sensitivity analyses stratified by group, (cross-sectional vs. longitudinal studies), revealed that 12.1%, (5.5-24.6 95%CI) of individuals with ASD have catatonia at baseline, and 8.0% (2.4-23.4 95%CI) of subjects diagnosed with ASD develop catatonic symptoms during the follow-up period, showing not differences depending on the type of study (p=0.801). |

**eFigure 1:** Meta-funnel results for Catatonia in ASD.

**eTable 5.** Meta-analytical results. % Catatonia in ASD.

| N studies | Total sample | % | 95%CI | Z | P value | Q | df | I^2^ | P | Egger test | p |
| --- | --- | --- | --- | --- | --- | --- | --- | --- | --- | --- | --- |
| 7 | 969 | 10.4 | 5.8-18.0 | -6.652 | 0.000 | 36.597 | 6 | 83.605 | 0.000 | 0.018 | 0.986 |

**eTable 6:** Risk of bias (quality assessment) using modified Newcastle Ottawa Scale for longitudinal studies.

| Study | Sample Representativeness | Exposure method | Groups match and adjustment | Assessment of outcome | Follow-up period | Losses to follow-up | Total score |
| --- | --- | --- | --- | --- | --- | --- | --- |
| Billstedt 2005 (5) | 1 | 1 | 1 | 1 | 1 | 1 | 6 |
| Hutton 2008 (6) | 1 | 1 | 0 | 1 | 1 | 1 | 5 |
| Ohta 2006 (7) | 1 | 0 | 0 | 1 | 1 | 1 | 4 |
| Wachtel 2017 (8) | 1 | 0 | 0 | 1 | 1 | 1 | 4 |
| Wachtel 2018 (9) | 1 | 0 | 0 | 1 | 1 | 1 | 4 |
| Wachtel 2019 (10) | 1 | 0 | 1 | 1 | 1 | 1 | 5 |

**eTable 7:** Risk of bias (quality assessment) using modified Newcastle Ottawa Scale for cross-sectional studies.

| Study | Sample Representativeness | Sample Size | Characteristics responders / non responders | Ascertainment of exposure | Assessment of outcome | Outcome reported | Total score |
| --- | --- | --- | --- | --- | --- | --- | --- |
| Breen 2017 (11) | 1 | 1 | 0 | 1 | 1 | 1 | 5 |
| Ghaziuddin 2012 (12) | 1 | 1 | 1 | 1 | 2 | 1 | 7 |
| Hare 2019 (13) | 1 | 1 | 0 | 1 | 1 | 1 | 5 |
| Périsse 2010 (14) | 2 | 1 | 0 | 1 | 1 | 1 | 6 |
| Wing 2000 (15) | 2 | 1 | 0 | 1 | 2 | 1 | 7 |
| Wing 2006 (16) | 2 | 1 | 0 | 1 | 2 | 1 | 7 |

**eTable 8.** Symptom overlap between Catatonia and ASD

| Symptoms | ASD | Catatonia | Comment |
| --- | --- | --- | --- |
| Symptoms present before 3 years (early developmental period) | +++ | - | In ASD, symptoms typically appear during pre-school years. |
| Emergence of symptoms in adolescence or young adulthood | - | ++ | Not seen in ASD. Typically reported in catatonia. |
| Deficits in social-emotional reciprocity | +++ | ++ | Core ASD symptom. Frequent in catatonia. |
| Deficits in nonverbal communicative behaviors | +++ | ++ | Core ASD symptom. Frequent in catatonia. |
| Deficits in developing, maintaining, and understanding relationships | +++ | ++ | Core ASD symptom. Frequent in catatonia. |
| Stereotyped or repetitive motor movements, use of objects, or speech | +++ | +++ | Core symptom in ASD and catatonia. |
| Insistence on sameness, inflexible adherence to routines, or ritualized patterns of behavior | +++ | + | Core symptom in ASD. Rituals may occasionally appear in catatonia. |
| Highly restricted, fixated interests | +++ | - | Core symptom in ASD. Not typically reported in catatonia. |
| Hyper- or hyporeactivity to sensory input or unusual interest in sensory aspects of environment | +++ | - | Core symptom in ASD. Does not typically appear in catatonia |
| Unusual thought content | + | ++ | May appear in ASD associated with a circumscribed intense interest. It can occur in some catatonic subtypes. |
| Thought interference | - | ++ | May appear in some catatonic subtypes. Not typical in ASD. |
| Thought perseveration | ++ | + | May appear in ASD and some catatonic subtypes. |
| Thought pressure | - | + | May appear in some catatonic subtypes. Not typical in ASD. |
| Thought blockages | - | + | May appear in some catatonic subtypes. Not typical in ASD. |
| Suspiciousness | + | + | May appear in ASD, particularly in those who misconstrue social cues, and in some catatonic subtypes. |
| Unstable ideas of reference | - | + | May appear in some catatonic subtypes. Not typical in ASD. |
| Perceptual abnormalities | + | + | May appear in ASD as part of ‘imaginary friends’ since early life and not a new phenomenon. It can occur in some catatonic subtypes. |
| Visual perception disturbances | + | + | May appear in ASD due to sensory issues and in some catatonic subtypes. |
| Acoustic perception disturbances | + | + | May appear in ASD due to sensory hyperresponsivity but is not a new-onset phenomenon. It can occur in some catatonic subtypes. |
| Decreased ability to discriminate between ideas, perception, fantasy and true memories | + | + | May appear in some catatonic subtypes and ASD. |
| Derealisation | - | - | Not typical in ASD or catatonia. |
| Disorganized communication | + | + | May appear in ASD and in some catatonic subtypes. |
| Disturbance of expressive speech | ++ | ++ | May appear in ASD and in some catatonic subtypes. |
| Disturbance of receptive speech | +++ | ++ | Core symptom in ASD. May appear in some catatonic subtypes. |
| Grandiose ideas | - | - | Not typically present in ASD or catatonia. |
| Disturbances of abstract thinking | +++ | - | Core symptom in ASD. Not typical in catatonia. |
| Inability to divide attention | ++ | + | May appear in ASD and catatonia. |
| Captivation of attention by details of the visual field | +++ | - | Core symptom in ASD. Not typical in catatonia. |
| Mannerism | +++ | +++ | Core symptom in ASD and catatonia. |
| Stereotypy | +++ | +++ | Core symptom in ASD and catatonia. |
| Agitation not influenced by external stimuli | ++ | +++ | Core symptom in catatonia. Frequently reported in ASD. |
| Grimacing | + | +++ | Core symptom in catatonia. May appear in ASD. |
| Echolalia | +++ | +++ | Core symptom in ASD and catatonia. |
| Echopraxia | + | +++ | Core symptom in catatonia. Frequently reported in ASD. |
| Stupor | - | +++ | Core symptom catatonia. Not reported in ASD. |
| Catalepsy | - | +++ | Core symptom catatonia. Not typically reported in ASD. |
| Waxy flexibility | - | +++ | Core symptom catatonia. Not typically reported in ASD. |
| Mutism | - | +++ | Core symptom catatonia. Not typically reported in ASD. |
| Negativism | - | +++ | Core symptom catatonia. Not typically reported in ASD. |
| Posturing | - | +++ | Core symptom catatonia. Not typically reported in ASD. |

**REFERENCES**

1. American Psychiatric Association. Diagnostic and statistical manual of mental disorders: DSM-IV-TR (text revision). American Journal of Psychiatry. 2000.

2. American Psychiatric Association. American Psychiatric Association, 2013. Diagnostic and statistical manual of mental disorders (5th ed.). American Journal of Psychiatry. 2013.

3. World Health Organization. The ICD-10 classification of mental and behavioural disorders: Diagnostic criteria for research. The ICD-10 classification of mental and behavioural disorders: Diagnostic criteria for research. 1993.

4. Stroup DF, Berlin JA, Morton SC, Olkin I, Williamson GD, Rennie D, et al. Meta-analysis of observational studies in epidemiology: A proposal for reporting. J Am Med Assoc. 2000;

5. Billstedt E, Gillberg C, Gillberg C. Autism after adolescence: Population-based 13- to 22-year follow-up study of 120 individuals with autism diagnosed in childhood. J Autism Dev Disord. 2005;

6. Hutton J, Goode S, Murphy M, Le Couteur A, Rutter M. New-onset psychiatric disorders in individuals with autism. Autism. 2008;12(4):373–90.

7. Ohta M, Kano Y, Nagai Y. Catatonia in Individuals With Autism Spectrum Disorders in Adolescence and Early Adulthood: A Long-term Prospective Study. International Review of Neurobiology. 2006.

8. Wachtel LE. Acute And Maintenance Electroconvulsive Therapy For Catatonia In Autism Spectrum Disorders. J Am Acad Child Adolesc Psychiatry. 2017;56(10S):70.3 — 71.0.

9. Wachtel LE. The multiple faces of catatonia in autism spectrum disorders: descriptive clinical experience of 22 patients over 12 years. Eur Child Adolesc Psychiatry. 2018;

10. Wachtel LE. Treatment of catatonia in autism spectrum disorders. Acta Psychiatr Scand. 2018;139(1):46–55.

11. Breen J, Hare DJ. The nature and prevalence of catatonic symptoms in young people with autism. J Intellect Disabil Res. 2017;

12. Ghaziuddin N, Dhossche D, Marcotte K. Retrospective chart review of catatonia in child and adolescent psychiatric patients. Acta Psychiatr Scand. 2012;

13. Hare D, Breen J, Bell L, Amoaka A, Oliver C, Moss J, et al. Assessment Of Attenuated Behaviour [Catatonia] In Idiopathic And Syndromic Autism. J Intellect Disabil Res. 2019;

14. Périsse D, Amiet C, Consoli A, Thorel MV, Gourfinkel-An I, Bodeau N, et al. Risk factors of acute behavioral regression in psychiatrically hospitalized adolescents with autism. J Can Acad Child Adolesc Psychiatry. 2010;19(2):100–8.

15. Wing L, Shah A. Catatonia in autistic spectrum disorders. Br J Psychiatry. 2000;

16. Wing L, Shah A. A Systematic Examination of Catatonia-like Clinical Pictures in Autism Spectrum Disorders. International Review of Neurobiology. 2006.
